# Redetermination and absolute configuration of berkeleydione

**DOI:** 10.1107/S2056989015003965

**Published:** 2015-03-21

**Authors:** Andrea Stierle, Donald Stierle, Daniel Decato

**Affiliations:** a Department of Biological and Pharmaceutical Sciences, University of Montana, 32 Campus Dr., Missoula, Montana 59812, USA

**Keywords:** crystal structure, absolute structure, resonant scattering, Berkeley pit, helicity rule

## Abstract

The crystal structure of the title compound, berkeleydione [systematic name; (5a*S*,7*R*,9*S*,11*R*,11a*S*)-methyl 9-hy­droxy-1,1,5,7,9,11a-hexa­methyl-14-methyl­idene-3,8,10-trioxo-1,3,4,5a,6,7,8,9,10,11,11a,12-dodeca­hydro-7,11-methano­cycloocta­[4,5]cyclo­hepta­[1,2-*c*]pyran-11-carboxyl­ate], C_26_H_32_O_7_, has been reported previously [Stierle *et al.* (2004 ▸). *Org. Lett.* **6**, 1049–1052]. However, the absolute configuration could not be determined from the data collected with Mo *K*α radiation and has now been determined by refinement of the Flack parameter with data collected using Cu *K*α radiation. It is in agreement with the previous circular dichroism assignment, and the crystal packing is similar to that described previously.

## Related literature   

For further information on the isolation and properties of berkeleydione and related compounds, see: Stierle *et al.* (2004 ▸, 2011 ▸). For the previous NMR and circular dichroism structure determination, see: Stierle *et al.* (2004 ▸).
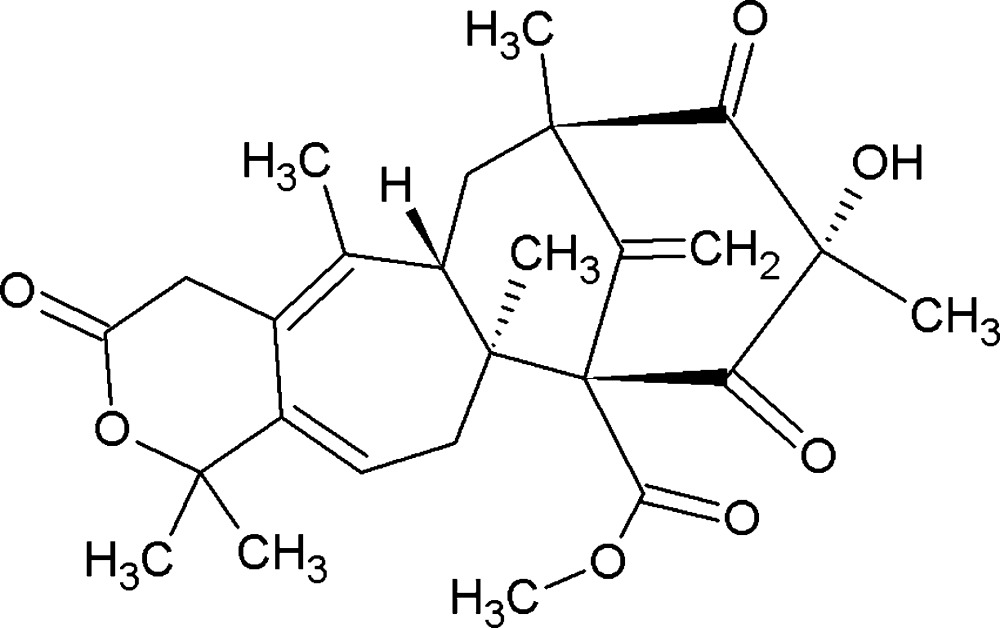


## Experimental   

### Crystal data  

C_26_H_32_O_7_
*M* *_r_* = 456.51
Orthorhombic, 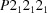

*a* = 9.1832 (6) Å
*b* = 14.5805 (9) Å
*c* = 17.5148 (11) Å
*V* = 2345.2 (3) Å^3^
*Z* = 4
Cu *K*α radiation
μ = 0.77 mm^−1^
*T* = 100 K
0.1 × 0.1 × 0.1 mm

### Data collection  

Bruker D8 Venture diffractometer
Absorption correction: multi-scan (*SADABS*; Bruker, 2009 ▸) *T*_min_ = 0.646, *T*_max_ = 0.754
39170 measured reflections
4814 independent reflections
4533 reflections with *I* > 2σ(*I*)
*R*_int_ = 0.051

### Refinement  

*R*[*F* ^2^ > 2σ(*F* ^2^)] = 0.034
*wR*(*F* ^2^) = 0.091
*S* = 1.06
4814 reflections
309 parameters
H atoms treated by a mixture of independent and constrained refinement
Δρ_max_ = 0.20 e Å^−3^
Δρ_min_ = −0.19 e Å^−3^
Absolute structure: Flack *x* determined using 1914 quotients [(*I* ^+^)−(*I* ^−^)]/[(*I* ^+^)+(*I* ^−^)] (Parsons *et al.*, 2013 ▸)
Absolute structure parameter: 0.07 (7)

### 

Data collection: *APEX2* (Bruker, 2009 ▸); cell refinement: *SAINT* (Bruker, 2009 ▸); data reduction: *SAINT*; program(s) used to solve structure: *SHELXS97* (Sheldrick, 2008 ▸); program(s) used to refine structure: *SHELXL2014* (Sheldrick, 2015 ▸); molecular graphics: *OLEX2* (Dolomanov *et al.*, 2009 ▸); software used to prepare material for publication: *OLEX2*.

## Supplementary Material

Crystal structure: contains datablock(s) I. DOI: 10.1107/S2056989015003965/su5086sup1.cif

Structure factors: contains datablock(s) I. DOI: 10.1107/S2056989015003965/su5086Isup2.hkl

**Fig. 1.**
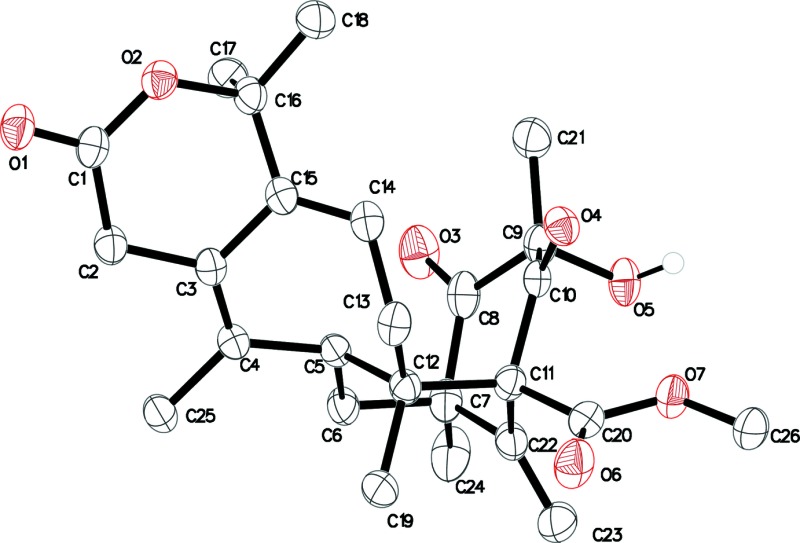
Molecular structure of the title compound with atom labelling. Displacement ellipsoides aredrawn at the 50% probability level. Hydrogen atoms have been omitted for clarity.

. DOI: 10.1107/S2056989015003965/su5086fig1.tif
Mol­ecular structure of the title compound with atom labelling. Displacement ellipsoides aredrawn at the 50% probability level. Hydrogen atoms have been omitted for clarity.

CCDC reference: 1051259

Additional supporting information:  crystallographic information; 3D view; checkCIF report

## supplementary crystallographic information

### S1. Synthesis and crystallization

Clear prisms of the title compound were grown by slow evaporation of a solution in water and methanol at 245 K.

### S2. Refinement

All the H atoms were located in difference Fourier maps and the hydroxyl H atom was freely refined. The C-bound H atoms were included in calculated positions and refined using a riding model: C—H = 0.98 - 1.00 Å with U_iso_(H) = 1.5U_eq_(C) for the methyl H atoms and = 1.2U_eq_(C) for the other H atoms.

### S3. Comment

The structure of berkeleydione, determined by detailed analysis of MS and NMR data has been reported (Stierle *et al.*, 2004). The X-ray structure was also determined but the absolute configuration could not be determined from the MoKα data collected. The helicity rule of circular dichroism for *cisoid* homoannular dienes (Stierle *et al.*, 2011) was applied to determine the absolute configuration of berkeleydione. The absolute configuration has now been determined by X-ray by refinement of the Flack parameter with data collected using CuKα radiation. This absolute configuration was shown to be the same as that determined with the helicity rule.

### Crystal data

**Table:**

C_26_H_32_O_7_	*D*_x_ = 1.293 Mg m^−^^3^
*M**_r_* = 456.51	Cu *K*α radiation, λ = 1.54178 Å
Orthorhombic, *P*2_1_2_1_2_1_	Cell parameters from 9233 reflections
*a* = 9.1832 (6) Å	θ = 3.9–74.7°
*b* = 14.5805 (9) Å	µ = 0.77 mm^−^^1^
*c* = 17.5148 (11) Å	*T* = 100 K
*V* = 2345.2 (3) Å^3^	Prism, colourless
*Z* = 4	0.1 × 0.1 × 0.1 mm
*F*(000) = 976	

### Data collection

**Table:**

Bruker D8 Venture diffractometer	4814 independent reflections
Radiation source: microfocus sealed X-ray tube, Incoatec Iµus	4533 reflections with *I* > 2σ(*I*)
Double Bounce Multilayer Mirror monochromator	*R*_int_ = 0.051
Detector resolution: 10.5 pixels mm^-1^	θ_max_ = 74.8°, θ_min_ = 4.0°
ω and φ scans	*h* = −11→11
Absorption correction: multi-scan (*SADABS*; Bruker, 2009)	*k* = −18→18
*T*_min_ = 0.646, *T*_max_ = 0.754	*l* = −21→21
39170 measured reflections	

### Refinement

**Table:**

Refinement on *F*^2^	Secondary atom site location: difference Fourier map
Least-squares matrix: full	Hydrogen site location: mixed
*R*[*F*^2^ > 2σ(*F*^2^)] = 0.034	H atoms treated by a mixture of independent and constrained refinement
*wR*(*F*^2^) = 0.091	*w* = 1/[σ^2^(*F*_o_^2^) + (0.0569*P*)^2^ + 0.2309*P*] where *P* = (*F*_o_^2^ + 2*F*_c_^2^)/3
*S* = 1.06	(Δ/σ)_max_ < 0.001
4814 reflections	Δρ_max_ = 0.20 e Å^−^^3^
309 parameters	Δρ_min_ = −0.19 e Å^−^^3^
0 restraints	Absolute structure: Flack *x* determined using 1914 quotients [(*I*^+^)-(*I*^-^)]/[(*I*^+^)+(*I*^-^)] (Parsons *et al.*, 2013)
Primary atom site location: structure-invariant direct methods	Absolute structure parameter: 0.07 (7)

### Special details

**Table:**

Geometry. All e.s.d.'s (except the e.s.d. in the dihedral angle between two l.s. planes) are estimated using the full covariance matrix. The cell e.s.d.'s are taken into account individually in the estimation of e.s.d.'s in distances, angles and torsion angles; correlations between e.s.d.'s in cell parameters are only used when they are defined by crystal symmetry. An approximate (isotropic) treatment of cell e.s.d.'s is used for estimating e.s.d.'s involving l.s. planes.

### Fractional atomic coordinates and isotropic or equivalent isotropic displacement parameters (Å^2^)

**Table:**

	*x*	*y*	*z*	*U*_iso_*/*U*_eq_	
O1	0.83088 (19)	0.23759 (10)	0.57687 (9)	0.0347 (4)	
O2	0.89494 (17)	0.30771 (9)	0.47209 (8)	0.0285 (3)	
O3	0.8155 (2)	0.76251 (12)	0.40185 (9)	0.0419 (4)	
O4	0.66301 (16)	0.56275 (9)	0.22151 (8)	0.0281 (3)	
O5	0.69193 (19)	0.77820 (9)	0.23618 (9)	0.0317 (3)	
O6	0.26938 (18)	0.55485 (13)	0.23397 (10)	0.0431 (4)	
O7	0.40659 (17)	0.65832 (10)	0.17368 (9)	0.0325 (3)	
C1	0.8103 (2)	0.30106 (13)	0.53388 (11)	0.0263 (4)	
C2	0.6911 (2)	0.36998 (14)	0.54887 (11)	0.0283 (4)	
H2A	0.7163	0.4042	0.5959	0.034*	
H2B	0.6000	0.3359	0.5593	0.034*	
C3	0.6607 (2)	0.43885 (13)	0.48639 (11)	0.0242 (4)	
C4	0.5814 (2)	0.51480 (14)	0.49700 (11)	0.0256 (4)	
C5	0.5717 (2)	0.58423 (13)	0.43178 (11)	0.0231 (4)	
H5A	0.6701	0.5848	0.4074	0.028*	
C6	0.5449 (2)	0.68248 (14)	0.45934 (11)	0.0286 (4)	
H6A	0.6173	0.6985	0.4990	0.034*	
H6B	0.4467	0.6868	0.4824	0.034*	
C7	0.5568 (3)	0.75115 (14)	0.39198 (11)	0.0300 (5)	
C8	0.7131 (3)	0.74107 (14)	0.36265 (12)	0.0303 (5)	
C9	0.7363 (2)	0.70236 (14)	0.28192 (11)	0.0267 (4)	
C10	0.6291 (2)	0.62327 (12)	0.26507 (11)	0.0223 (4)	
C11	0.4753 (2)	0.62691 (14)	0.30026 (11)	0.0244 (4)	
C12	0.4637 (2)	0.55420 (14)	0.36813 (11)	0.0252 (4)	
C13	0.5029 (2)	0.45512 (14)	0.34211 (12)	0.0281 (4)	
H13A	0.4463	0.4116	0.3737	0.034*	
H13B	0.4700	0.4475	0.2886	0.034*	
C14	0.6610 (2)	0.42778 (12)	0.34624 (11)	0.0256 (4)	
H14	0.7127	0.4174	0.3001	0.031*	
C15	0.7308 (2)	0.41761 (12)	0.41267 (12)	0.0240 (4)	
C16	0.8869 (2)	0.38926 (13)	0.42057 (11)	0.0272 (4)	
C17	0.9756 (3)	0.46460 (15)	0.45895 (13)	0.0324 (5)	
H17A	0.9326	0.4792	0.5087	0.049*	
H17B	0.9752	0.5195	0.4267	0.049*	
H17C	1.0760	0.4435	0.4661	0.049*	
C18	0.9605 (3)	0.35696 (16)	0.34803 (13)	0.0377 (5)	
H18A	1.0577	0.3334	0.3602	0.057*	
H18B	0.9692	0.4084	0.3123	0.057*	
H18C	0.9023	0.3082	0.3246	0.057*	
C19	0.3075 (2)	0.55189 (18)	0.39963 (13)	0.0366 (5)	
H19A	0.3050	0.5147	0.4462	0.055*	
H19B	0.2423	0.5250	0.3614	0.055*	
H19C	0.2757	0.6145	0.4114	0.055*	
C20	0.3701 (2)	0.60662 (14)	0.23390 (13)	0.0290 (4)	
C21	0.8924 (2)	0.67381 (16)	0.26896 (14)	0.0351 (5)	
H21A	0.9055	0.6559	0.2155	0.053*	
H21B	0.9159	0.6218	0.3022	0.053*	
H21C	0.9572	0.7253	0.2808	0.053*	
C22	0.4455 (2)	0.72279 (14)	0.33273 (11)	0.0284 (4)	
C23	0.3275 (3)	0.77049 (16)	0.31622 (13)	0.0399 (5)	
H23A	0.3083	0.8264	0.3422	0.048*	
H23B	0.2618	0.7489	0.2784	0.048*	
C24	0.5349 (3)	0.84908 (15)	0.42152 (14)	0.0449 (6)	
H24A	0.5350	0.8919	0.3784	0.067*	
H24B	0.6141	0.8649	0.4566	0.067*	
H24C	0.4415	0.8531	0.4484	0.067*	
C25	0.5110 (3)	0.53681 (16)	0.57273 (12)	0.0339 (5)	
H25A	0.5036	0.4808	0.6034	0.051*	
H25B	0.4134	0.5619	0.5640	0.051*	
H25C	0.5703	0.5821	0.6000	0.051*	
C26	0.3148 (3)	0.64775 (18)	0.10765 (13)	0.0379 (5)	
H26A	0.3550	0.6834	0.0652	0.057*	
H26B	0.2166	0.6698	0.1195	0.057*	
H26C	0.3104	0.5828	0.0933	0.057*	
H5	0.690 (3)	0.760 (2)	0.1879 (18)	0.048 (8)*	

### Atomic displacement parameters (Å^2^)

**Table:**

	*U*^11^	*U*^22^	*U*^33^	*U*^12^	*U*^13^	*U*^23^
O1	0.0492 (9)	0.0275 (7)	0.0272 (7)	0.0056 (7)	−0.0029 (7)	0.0036 (6)
O2	0.0348 (8)	0.0230 (6)	0.0278 (7)	0.0035 (6)	−0.0013 (6)	0.0001 (6)
O3	0.0490 (10)	0.0459 (9)	0.0308 (8)	−0.0174 (8)	−0.0085 (8)	−0.0017 (7)
O4	0.0343 (8)	0.0247 (6)	0.0252 (7)	−0.0003 (6)	0.0025 (6)	−0.0005 (6)
O5	0.0476 (9)	0.0231 (6)	0.0242 (7)	−0.0044 (6)	−0.0031 (7)	0.0044 (6)
O6	0.0364 (9)	0.0530 (10)	0.0400 (9)	−0.0150 (8)	−0.0122 (7)	0.0104 (8)
O7	0.0357 (8)	0.0360 (8)	0.0258 (7)	−0.0023 (7)	−0.0060 (7)	0.0046 (6)
C1	0.0337 (10)	0.0222 (8)	0.0230 (9)	−0.0024 (8)	−0.0059 (8)	−0.0010 (7)
C2	0.0351 (11)	0.0263 (9)	0.0235 (9)	0.0014 (8)	0.0006 (8)	0.0024 (7)
C3	0.0271 (10)	0.0242 (9)	0.0213 (9)	−0.0037 (7)	−0.0009 (7)	0.0016 (7)
C4	0.0268 (9)	0.0282 (9)	0.0218 (9)	−0.0006 (7)	0.0022 (8)	0.0030 (7)
C5	0.0247 (10)	0.0246 (9)	0.0200 (9)	0.0011 (7)	0.0026 (7)	0.0012 (7)
C6	0.0385 (11)	0.0274 (10)	0.0200 (9)	0.0069 (9)	0.0018 (8)	−0.0002 (8)
C7	0.0467 (13)	0.0212 (9)	0.0222 (9)	0.0053 (8)	−0.0017 (9)	−0.0012 (7)
C8	0.0443 (13)	0.0208 (8)	0.0256 (10)	−0.0071 (8)	−0.0054 (9)	0.0033 (7)
C9	0.0302 (10)	0.0260 (9)	0.0240 (9)	−0.0068 (8)	−0.0017 (8)	0.0043 (8)
C10	0.0274 (9)	0.0214 (8)	0.0181 (8)	−0.0011 (7)	−0.0015 (7)	0.0041 (7)
C11	0.0254 (9)	0.0236 (9)	0.0240 (9)	0.0004 (7)	−0.0015 (8)	0.0034 (7)
C12	0.0236 (9)	0.0282 (9)	0.0239 (9)	−0.0027 (8)	0.0005 (8)	0.0057 (7)
C13	0.0341 (11)	0.0235 (9)	0.0268 (10)	−0.0079 (8)	−0.0064 (8)	0.0033 (8)
C14	0.0376 (11)	0.0168 (8)	0.0225 (9)	−0.0007 (8)	0.0000 (8)	−0.0012 (7)
C15	0.0312 (10)	0.0178 (8)	0.0229 (9)	−0.0014 (7)	0.0021 (8)	0.0001 (7)
C16	0.0337 (11)	0.0229 (9)	0.0251 (9)	0.0024 (8)	0.0024 (8)	0.0029 (7)
C17	0.0297 (10)	0.0307 (10)	0.0369 (11)	−0.0045 (8)	−0.0003 (9)	0.0036 (9)
C18	0.0438 (13)	0.0372 (11)	0.0322 (11)	0.0140 (10)	0.0081 (10)	0.0020 (9)
C19	0.0253 (10)	0.0499 (13)	0.0347 (11)	−0.0023 (9)	0.0043 (9)	0.0141 (10)
C20	0.0286 (10)	0.0294 (9)	0.0290 (10)	0.0027 (8)	−0.0018 (9)	0.0028 (8)
C21	0.0295 (11)	0.0421 (11)	0.0337 (11)	−0.0071 (9)	−0.0010 (9)	0.0067 (9)
C22	0.0368 (12)	0.0259 (9)	0.0226 (9)	0.0043 (8)	0.0030 (8)	0.0029 (7)
C23	0.0466 (14)	0.0412 (12)	0.0320 (11)	0.0167 (11)	0.0006 (10)	0.0003 (9)
C24	0.0781 (18)	0.0246 (10)	0.0319 (12)	0.0116 (11)	−0.0072 (13)	−0.0039 (9)
C25	0.0394 (12)	0.0370 (11)	0.0254 (10)	0.0078 (9)	0.0084 (9)	0.0056 (9)
C26	0.0374 (12)	0.0503 (13)	0.0259 (10)	0.0068 (10)	−0.0070 (9)	0.0001 (9)

### Geometric parameters (Å, º)

**Table:**

O1—C1	1.208 (2)	C12—C13	1.557 (3)
O2—C1	1.336 (3)	C12—C19	1.537 (3)
O2—C16	1.494 (2)	C13—H13A	0.9900
O3—C8	1.206 (3)	C13—H13B	0.9900
O4—C10	1.207 (2)	C13—C14	1.507 (3)
O5—C9	1.425 (2)	C14—H14	0.9500
O5—H5	0.89 (3)	C14—C15	1.337 (3)
O6—C20	1.194 (3)	C15—C16	1.498 (3)
O7—C20	1.339 (3)	C16—C17	1.524 (3)
O7—C26	1.439 (3)	C16—C18	1.514 (3)
C1—C2	1.509 (3)	C17—H17A	0.9800
C2—H2A	0.9900	C17—H17B	0.9800
C2—H2B	0.9900	C17—H17C	0.9800
C2—C3	1.511 (3)	C18—H18A	0.9800
C3—C4	1.338 (3)	C18—H18B	0.9800
C3—C15	1.476 (3)	C18—H18C	0.9800
C4—C5	1.529 (3)	C19—H19A	0.9800
C4—C25	1.510 (3)	C19—H19B	0.9800
C5—H5A	1.0000	C19—H19C	0.9800
C5—C6	1.531 (3)	C21—H21A	0.9800
C5—C12	1.555 (3)	C21—H21B	0.9800
C6—H6A	0.9900	C21—H21C	0.9800
C6—H6B	0.9900	C22—C23	1.320 (3)
C6—C7	1.551 (3)	C23—H23A	0.9500
C7—C8	1.531 (3)	C23—H23B	0.9500
C7—C22	1.514 (3)	C24—H24A	0.9800
C7—C24	1.532 (3)	C24—H24B	0.9800
C8—C9	1.537 (3)	C24—H24C	0.9800
C9—C10	1.545 (3)	C25—H25A	0.9800
C9—C21	1.510 (3)	C25—H25B	0.9800
C10—C11	1.542 (3)	C25—H25C	0.9800
C11—C12	1.596 (3)	C26—H26A	0.9800
C11—C20	1.540 (3)	C26—H26B	0.9800
C11—C22	1.534 (3)	C26—H26C	0.9800
			
C1—O2—C16	121.19 (15)	C14—C13—H13B	108.0
C9—O5—H5	107.9 (19)	C13—C14—H14	118.9
C20—O7—C26	115.23 (17)	C15—C14—C13	122.23 (19)
O1—C1—O2	117.99 (19)	C15—C14—H14	118.9
O1—C1—C2	121.03 (19)	C3—C15—C16	113.24 (17)
O2—C1—C2	120.98 (17)	C14—C15—C3	121.92 (19)
C1—C2—H2A	108.1	C14—C15—C16	124.76 (19)
C1—C2—H2B	108.1	O2—C16—C15	108.84 (16)
C1—C2—C3	116.77 (17)	O2—C16—C17	106.30 (16)
H2A—C2—H2B	107.3	O2—C16—C18	103.70 (15)
C3—C2—H2A	108.1	C15—C16—C17	110.69 (17)
C3—C2—H2B	108.1	C15—C16—C18	115.82 (18)
C4—C3—C2	123.34 (18)	C18—C16—C17	110.82 (19)
C4—C3—C15	122.18 (18)	C16—C17—H17A	109.5
C15—C3—C2	114.43 (17)	C16—C17—H17B	109.5
C3—C4—C5	118.42 (17)	C16—C17—H17C	109.5
C3—C4—C25	122.07 (18)	H17A—C17—H17B	109.5
C25—C4—C5	119.37 (17)	H17A—C17—H17C	109.5
C4—C5—H5A	105.8	H17B—C17—H17C	109.5
C4—C5—C6	113.15 (16)	C16—C18—H18A	109.5
C4—C5—C12	112.74 (16)	C16—C18—H18B	109.5
C6—C5—H5A	105.8	C16—C18—H18C	109.5
C6—C5—C12	112.76 (16)	H18A—C18—H18B	109.5
C12—C5—H5A	105.8	H18A—C18—H18C	109.5
C5—C6—H6A	109.5	H18B—C18—H18C	109.5
C5—C6—H6B	109.5	C12—C19—H19A	109.5
C5—C6—C7	110.66 (16)	C12—C19—H19B	109.5
H6A—C6—H6B	108.1	C12—C19—H19C	109.5
C7—C6—H6A	109.5	H19A—C19—H19B	109.5
C7—C6—H6B	109.5	H19A—C19—H19C	109.5
C8—C7—C6	105.02 (17)	H19B—C19—H19C	109.5
C8—C7—C24	109.0 (2)	O6—C20—O7	123.4 (2)
C22—C7—C6	107.30 (18)	O6—C20—C11	127.3 (2)
C22—C7—C8	112.11 (16)	O7—C20—C11	109.24 (17)
C22—C7—C24	113.42 (19)	C9—C21—H21A	109.5
C24—C7—C6	109.61 (17)	C9—C21—H21B	109.5
O3—C8—C7	121.0 (2)	C9—C21—H21C	109.5
O3—C8—C9	120.7 (2)	H21A—C21—H21B	109.5
C7—C8—C9	118.28 (18)	H21A—C21—H21C	109.5
O5—C9—C8	101.10 (16)	H21B—C21—H21C	109.5
O5—C9—C10	106.84 (16)	C7—C22—C11	112.51 (17)
O5—C9—C21	113.64 (17)	C23—C22—C7	124.1 (2)
C8—C9—C10	111.18 (16)	C23—C22—C11	123.1 (2)
C21—C9—C8	111.79 (18)	C22—C23—H23A	120.0
C21—C9—C10	111.75 (17)	C22—C23—H23B	120.0
O4—C10—C9	120.13 (18)	H23A—C23—H23B	120.0
O4—C10—C11	120.94 (17)	C7—C24—H24A	109.5
C11—C10—C9	118.79 (16)	C7—C24—H24B	109.5
C10—C11—C12	109.63 (15)	C7—C24—H24C	109.5
C20—C11—C10	105.44 (16)	H24A—C24—H24B	109.5
C20—C11—C12	113.12 (16)	H24A—C24—H24C	109.5
C22—C11—C10	110.06 (16)	H24B—C24—H24C	109.5
C22—C11—C12	108.50 (16)	C4—C25—H25A	109.5
C22—C11—C20	110.07 (17)	C4—C25—H25B	109.5
C5—C12—C11	107.71 (15)	C4—C25—H25C	109.5
C5—C12—C13	108.88 (16)	H25A—C25—H25B	109.5
C13—C12—C11	112.50 (16)	H25A—C25—H25C	109.5
C19—C12—C5	110.12 (17)	H25B—C25—H25C	109.5
C19—C12—C11	110.13 (17)	O7—C26—H26A	109.5
C19—C12—C13	107.49 (18)	O7—C26—H26B	109.5
C12—C13—H13A	108.0	O7—C26—H26C	109.5
C12—C13—H13B	108.0	H26A—C26—H26B	109.5
H13A—C13—H13B	107.3	H26A—C26—H26C	109.5
C14—C13—C12	117.01 (16)	H26B—C26—H26C	109.5
C14—C13—H13A	108.0		
			
O1—C1—C2—C3	−171.76 (19)	C9—C10—C11—C20	131.44 (17)
O2—C1—C2—C3	7.3 (3)	C9—C10—C11—C22	12.8 (2)
O3—C8—C9—O5	−106.8 (2)	C10—C11—C12—C5	64.23 (19)
O3—C8—C9—C10	140.1 (2)	C10—C11—C12—C13	−55.8 (2)
O3—C8—C9—C21	14.4 (3)	C10—C11—C12—C19	−175.66 (17)
O4—C10—C11—C12	77.8 (2)	C10—C11—C20—O6	133.4 (2)
O4—C10—C11—C20	−44.2 (2)	C10—C11—C20—O7	−47.7 (2)
O4—C10—C11—C22	−162.91 (17)	C10—C11—C22—C7	−57.5 (2)
O5—C9—C10—O4	99.8 (2)	C10—C11—C22—C23	129.0 (2)
O5—C9—C10—C11	−76.0 (2)	C11—C12—C13—C14	88.1 (2)
C1—O2—C16—C15	−39.0 (2)	C12—C5—C6—C7	−58.2 (2)
C1—O2—C16—C17	80.2 (2)	C12—C11—C20—O6	13.6 (3)
C1—O2—C16—C18	−162.85 (18)	C12—C11—C20—O7	−167.50 (16)
C1—C2—C3—C4	−165.57 (19)	C12—C11—C22—C7	62.4 (2)
C1—C2—C3—C15	12.1 (3)	C12—C11—C22—C23	−111.0 (2)
C2—C3—C4—C5	173.63 (18)	C12—C13—C14—C15	67.9 (2)
C2—C3—C4—C25	−1.9 (3)	C13—C14—C15—C3	−4.4 (3)
C2—C3—C15—C14	137.95 (19)	C13—C14—C15—C16	179.03 (17)
C2—C3—C15—C16	−45.1 (2)	C14—C15—C16—O2	−125.66 (19)
C3—C4—C5—C6	−152.62 (19)	C14—C15—C16—C17	117.9 (2)
C3—C4—C5—C12	77.9 (2)	C14—C15—C16—C18	−9.4 (3)
C3—C15—C16—O2	57.5 (2)	C15—C3—C4—C5	−3.8 (3)
C3—C15—C16—C17	−59.0 (2)	C15—C3—C4—C25	−179.4 (2)
C3—C15—C16—C18	173.82 (17)	C16—O2—C1—O1	−173.54 (18)
C4—C3—C15—C14	−44.4 (3)	C16—O2—C1—C2	7.3 (3)
C4—C3—C15—C16	132.5 (2)	C19—C12—C13—C14	−150.45 (18)
C4—C5—C6—C7	172.39 (18)	C20—C11—C12—C5	−178.42 (16)
C4—C5—C12—C11	−174.52 (16)	C20—C11—C12—C13	61.6 (2)
C4—C5—C12—C13	−52.3 (2)	C20—C11—C12—C19	−58.3 (2)
C4—C5—C12—C19	65.4 (2)	C20—C11—C22—C7	−173.30 (17)
C5—C6—C7—C8	−61.0 (2)	C20—C11—C22—C23	13.2 (3)
C5—C6—C7—C22	58.4 (2)	C21—C9—C10—O4	−25.1 (3)
C5—C6—C7—C24	−178.0 (2)	C21—C9—C10—C11	159.17 (17)
C5—C12—C13—C14	−31.2 (2)	C22—C7—C8—O3	178.38 (19)
C6—C5—C12—C11	55.8 (2)	C22—C7—C8—C9	−1.9 (2)
C6—C5—C12—C13	178.09 (16)	C22—C11—C12—C5	−56.0 (2)
C6—C5—C12—C19	−64.3 (2)	C22—C11—C12—C13	−175.99 (17)
C6—C7—C8—O3	−65.4 (2)	C22—C11—C12—C19	64.1 (2)
C6—C7—C8—C9	114.26 (18)	C22—C11—C20—O6	−107.9 (3)
C6—C7—C22—C11	−62.5 (2)	C22—C11—C20—O7	71.0 (2)
C6—C7—C22—C23	110.9 (2)	C24—C7—C8—O3	52.0 (3)
C7—C8—C9—O5	73.5 (2)	C24—C7—C8—C9	−128.35 (18)
C7—C8—C9—C10	−39.6 (2)	C24—C7—C22—C11	176.34 (19)
C7—C8—C9—C21	−165.25 (18)	C24—C7—C22—C23	−10.3 (3)
C8—C7—C22—C11	52.3 (2)	C25—C4—C5—C6	23.1 (3)
C8—C7—C22—C23	−134.3 (2)	C25—C4—C5—C12	−106.4 (2)
C8—C9—C10—O4	−150.80 (18)	C26—O7—C20—O6	0.2 (3)
C8—C9—C10—C11	33.5 (2)	C26—O7—C20—C11	−178.71 (17)
C9—C10—C11—C12	−106.49 (19)		
