# Supplementary figures and images for: Redetermination and absolute configuration of berkeleydione
Source: Acta Crystallogr E Crystallogr Commun. 2015 Mar 21;71(Pt 4):o248. doi: 10.1107/S2056989015003965 (PMC4438833; doi:10.1107/S2056989015003965)

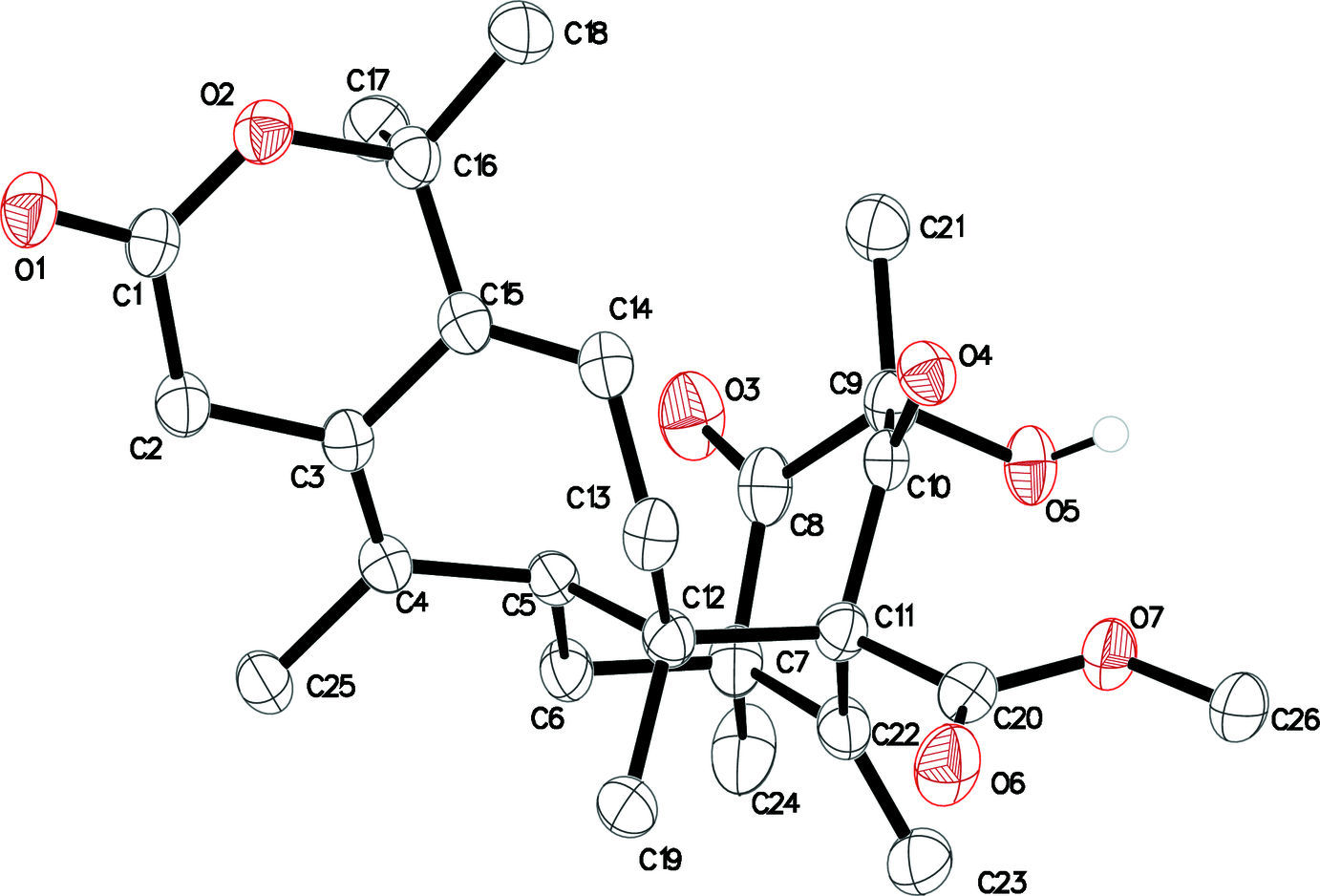

Supplement: Supplementary file 3 [file e-71-0o248-fig1.tif]
